# Unlocking the potential: current landscape and future directions of immunotherapy in gastric cancer

**DOI:** 10.3389/fimmu.2026.1742723

**Published:** 2026-05-12

**Authors:** Yingfei Zhou, Luling Wei, Luoyang Wang, Bei Zhang, Jie Liang

**Affiliations:** 1 Department of Clinical Medicine, Qingdao University, Qingdao, China; 2 Department of Immunology, School of Basic Medicine, Qingdao University, Qingdao, China

**Keywords:** biomarker, clinical trial, gastric cancer, immunotherapy, molecular target

## Abstract

Gastric cancer (GC) ranks as the third leading cause of cancer-related mortality worldwide, and its management remains formidable. Immunotherapy has been highly praised for its remarkable efficacy and acceptable toxicity, and its development has outpaced that of traditional therapies. However, molecular heterogeneity and the immunosuppressive tumor immune microenvironment (TIME) have hindered the treatment response of a considerable number of patients. This review synthesizes the latest therapeutic advances, spanning immune-checkpoint inhibitors (ICIs), adoptive cell therapy (ACT), monoclonal antibodies and antibody drug conjugates (ADCs), cancer vaccines, tumor-infiltrating lymphocyte (TIL) therapy, and CAR-T cells therapy. Emerging strategies such as RNA interference nano-delivery systems, immune adjuvants, and microbiota modulation are constantly evolving to transform “cold” tumors into “hot” tumors. Persistent challenges include primary resistance, immune-related adverse events (irAEs) and antigenic heterogeneity, underscoring the imperative for refined patient stratification. Classical biomarkers such as PD-L1 expression, tumor mutational burden (TMB), mismatch-repair status, Epstein–Barr virus (EBV) positivity and circulating tumor DNA (ctDNA) all demonstrate predictive value but remain constrained by spatial heterogeneity and temporal dynamics. Consequently, we highlight emerging biomarkers that integrate metabolic, epigenetic and cell-death signatures, providing a roadmap for precision immunotherapy and continuous optimization of GC treatment algorithms.

## Graphical Abstract

**Figure:**
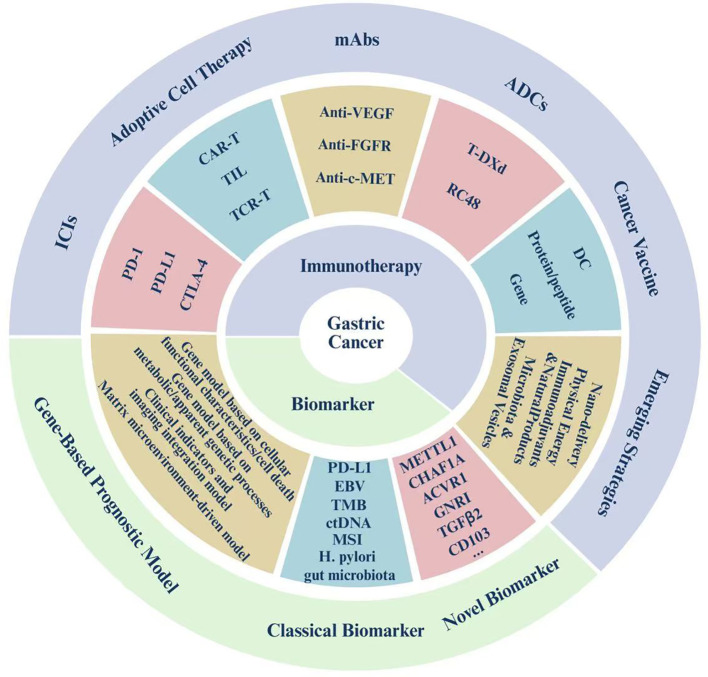
Immune Therapy Strategies and Biomarkers for Gastric Cancer. This figure focuses on gastric cancer and systematically presents the current and emerging immunotherapy directions: The upper ring covers emerging immunotherapy strategies such as immune checkpoint inhibitors, adoptive cell therapy, monoclonal antibodies, antibody-drug conjugates, cancer vaccines, and nanodelivery; the lower ring summarizes classic biomarkers (such as PD-L1, EBV, ctDNA), new molecular biomarkers (such as METTL1, CHAF1A), and gene prognostic models constructed based on cell characteristics, gene expression, and tumor microenvironment, comprehensively presenting the core technologies and biomarker systems that guide precise diagnosis and treatment of gastric cancer.

## 1 Introduction

Gastric cancer (GC) represents the sixth most common malignancy and the third leading cause of cancer-related mortality worldwide (1). The incidence rate shows significant regional and demographic variations, which are caused by factors that cannot be changed, such as age, gender, and race, as well as modifiable exposure factors such as tobacco consumption, nitrate/nitrite-rich diets and most notably Helicobacter pylori (H. pylori) infection. The current standard-of-care paradigm encompasses surgical resection, cytotoxic chemotherapy, and radiotherapy. Although gastrectomy (subtotal or total) remains the cornerstone of curative intent for localized GC, its therapeutic window narrows substantially in advanced disease and is invariably accompanied by considerable functional morbidity (2, 3). Moreover, adjuvant or neoadjuvant chemoradiation regimens, despite their integral role in multimodal management, frequently fail to eradicate micrometastatic disease because of tumor heterogeneity and intrinsic resistance mechanisms. Chemotherapeutic strategies have evolved from traditional DNA-damaging agents to targeted inhibitors of DNA replication and mitotic enzymes; however, their clinical utility remains constrained by variable response rates and dose-dependent systemic toxicity. Collectively, these challenges highlight the critical need for more precise and effective therapeutic strategies in GC management (4).

**Figure 1:**
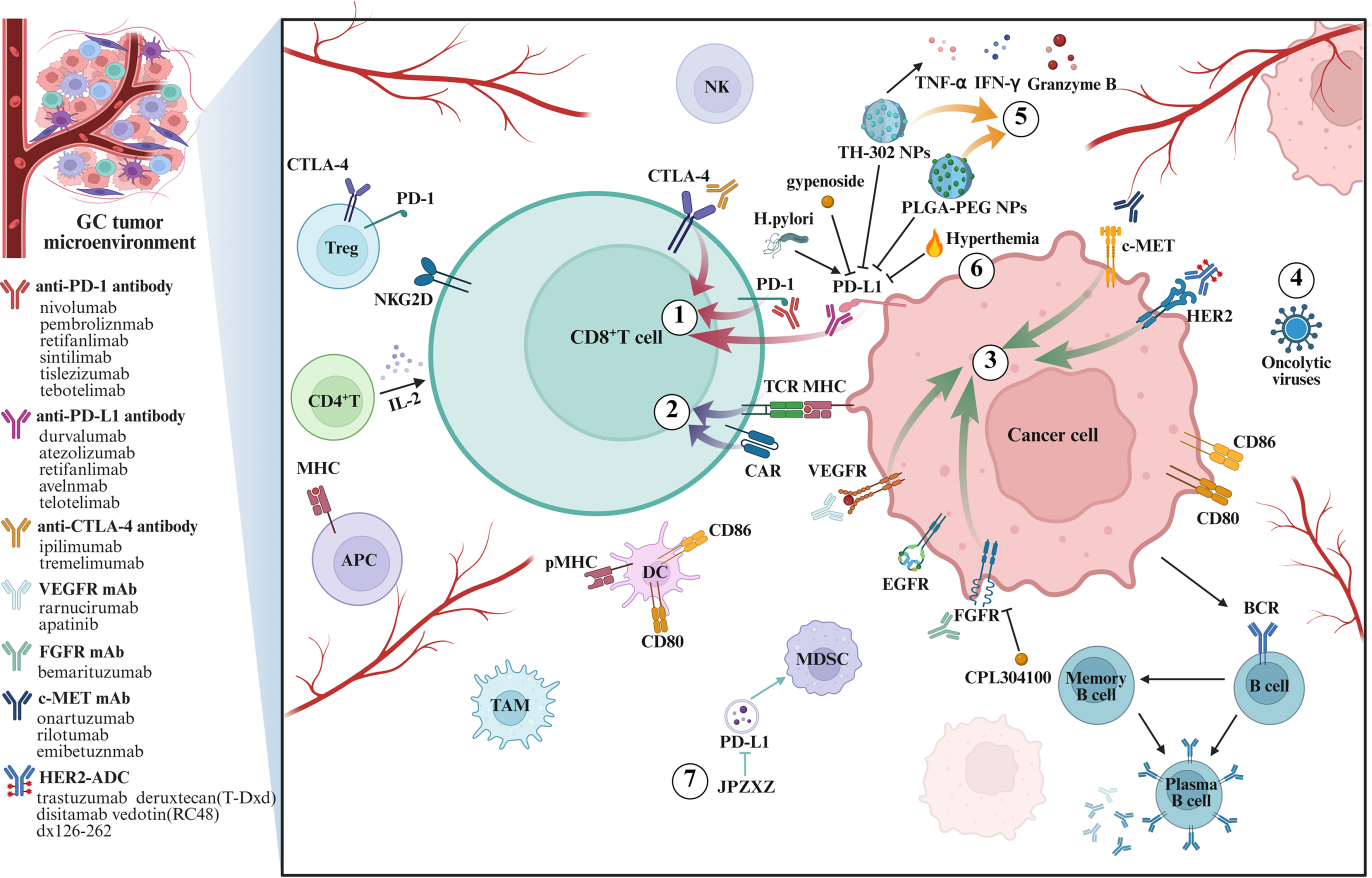
Key targets and signaling pathways of GC immunotherapy. In TME, tumor cells and immune cells interact through cell surface molecules. Different treatment methods target different molecules to exert regulatory effects on tumor cells and immune cells, thereby inhibiting GC. The therapies shown in the picture include: (1) ICIs; (2) Adoptive cell therapy; (3) The mAbs and ADCs; (4) Oncolytic virus therapy; (5) Precise regulation of nano-delivery systems; (6) Immunological synergistic effect of physical energy therapy; (7) Microenvironment regulation by immunoadjuvants and natural products.

Conventional therapies for GC exhibit limited and heterogeneous efficacy and are invariably accompanied by substantial toxicity. In contrast, based on the deepening understanding of the tumor microenvironment (TME), immunotherapy for GC has been gradually applied in clinical practice, it can provide therapeutic options for numerous GC patients who are refractory to conventional approaches, holds advantages that radiotherapy and chemotherapy do not possess in the treatment of advanced and recurrent GC, such as prolonging the survival time of patients and generating more sustained immune response effects, thereby offering new hope to patients with GC (5). Current immunotherapeutic strategies for GC encompass several modalities (**Figure 1**), including immune checkpoint inhibitors (ICIs) (e.g., anti-programmed cell death protein 1 (PD-1), anti-programmed death-ligand 1 (PD-L1), and anti-cytotoxic T-lymphocyte-associated protein 4 (CTLA-4) monoclonal antibodies (mAbs)), adoptive cell therapies (ACTs) (such as tumor-infiltrating lymphocyte (TIL) therapy, engineered T cell receptor (TCR)-T therapy, natural killer (NK) cell therapy, and chimeric antigen receptor (CAR)-T cell therapy), mAbs and antibody-drug conjugates (ADCs) (targeting vascular endothelial growth factor (VEGF), fibroblast growth factor receptor (FGFR), c-mesenchymal-epithelial transition factor (c-MET), etc.), and cancer vaccines (6). Among these, the most significant clinical advancements have been achieved with PD-1/PD-L1 inhibitors, which have demonstrated remarkable efficacy in reshaping the therapeutic landscape of GC (7).

However, many types of cancer are described as “cold tumors”, where the infiltration of immune cells that kill the tumor is limited within the tumor, significantly reducing the efficacy of immune-checkpoint blockade (ICB) (8). Consequently, a substantial proportion of patients derive minimal or no clinical benefit from immunotherapy—a deficit that likely reflects our incomplete understanding of the GC-specific tumor immune microenvironment (TIME). Moreover, immune-related adverse events (irAEs) remain frequent and can further curtail therapeutic utility (9). To optimize the efficacy of immunotherapy, we still need to explore immunotherapy combination strategies (7), such as immunotherapy combined with other therapies. By the way, the success of immunotherapy depends to a large extent on the TME and its interaction with the immune system. The activated immune phenotype is correlated with better survival rates, while the immunosuppressive state significantly impedes the effect of immunotherapy (10). Hence, the identification of biomarkers related to these interactions is expected to guide personalized treatment strategies, but finding the best predictive biomarkers for GC immunotherapy is also challenging because each biomarker has its limitations (11).

In this review, we first introduced the basic principles of immunotherapy, and then systematically evaluate recent advances in GC immunotherapy, with particular emphasis on ICIs, ACT, mAbs, and ADCs. We further delineate the mechanistic underpinnings of these modalities and critically examine current limitations, including primary and acquired resistance as well as irAEs.

However, from a clinical perspective, the key issue faced by oncologists is not merely what therapies are available, but which therapy should be prioritized for a specific patient, and how to identify patients who are suitable for treatment. To answer this question, we integrate established and emerging clinical biomarkers to refine patient stratification and inform rational combination strategies, thereby advancing precision immuno-oncology for GC.

## 2 Basic principles of immunotherapy

Immunotherapy has revolutionized the treatment paradigm for various malignancies; however, its efficacy is often limited by intrinsic or acquired resistance mechanisms, which may arise from the unique toxicity profiles and distinct biological pathways associated with different immunotherapeutic agents (12). The cellular foundation of immunotherapy relies heavily on TILs, which play a pivotal role in mediating antitumor immune responses. In highly immunogenic tumors, the cellular foundation relies on TIL, with “hot” tumors (>40% TIL density) showing superior response rates versus immunologically “cold” tumors (e.g., glioblastoma). Nevertheless, emerging therapeutic approaches are actively exploring methods to augment TIL recruitment and activation within these immunologically quiescent tumors, thereby converting them into “hot” tumors and reinvigorating antitumor immunity (13).

Although the immune system can initially monitor and recognize cancer cells, tumors eventually enter an immune escape state through immunoediting, exploiting the immune system to facilitate metastasis (4). Immune escape, also termed immune evasion or antigen escape, occurs when the host immune system fails to mount an effective response against malignant cells (14). The heterogeneous TME promotes immune evasion, leading to resistance to conventional and immunotherapies (9). The mechanism of tumor immune escape is complex, and cancer cells can escape immune surveillance through various mechanisms, such as defects in antigen presentation mechanisms, upregulation of negative regulatory pathways, and recruitment of immune-suppressive cell populations, thereby hindering the effector functions of immune cells and eliminating the anti-tumor immune response (15).

Tumor immune escape in GC arises through two interlocking layers of dysfunction. First, tumor cell-intrinsic mechanisms: These include loss of major histocompatibility complex (MHC) molecules or co-stimulatory signals, reduced immunogenicity of tumor-associated antigens (TAAs), and downregulation of antigen-processing and presentation-related genes. Second, the host immune apparatus is disabled by its failure to detect low-abundance TAAs during early tumorigenesis, as well as immunosuppression mediated by myeloid-derived suppressor cells (MDSCs), regulatory T cells (Tregs), and tumor-associated macrophages (TAMs), functional paralysis of antigen-presenting cells (APCs), and systemic T-cell exhaustion (4). These processes converge to extinguish effector immunity. Metabolic reprogramming lies at the core of this evasion. Within the GC TME, heightened glycolysis, lipogenesis, and amino-acid metabolism fuel neoplastic proliferation and migration while simultaneously rewiring immune-cell function and immune-molecule availability, thereby reinforcing immune privilege (16). Immune checkpoint molecules serve as pivotal mediators of tumor immune escape. Upon interaction with cancer cells, these checkpoints suppress T cell activation and amplify inhibitory signals through surface receptor engagement, thereby facilitating cancer progression and metastasis. Additionally, certain tumor cells can recruit and activate immunosuppressive leukocytes, fostering an immune-tolerant TME that attenuates antitumor immune responses. Consequently, antibody-mediated checkpoint inhibition has emerged as a promising strategy to counteract immune evasion and suppress tumor growth in malignancies such as GC (17).

Beyond these generalized mechanisms, GC exhibits pathogen-driven immune escape programs. H. pylori infection rapidly engages the JAK–STAT1 pathway, concomitantly up-regulating PD-L1; this dual switch disables cytotoxic CD8^+^ T-cell effector functions and fosters epithelial immune escape (18). Similarly, Epstein–Barr virus (EBV) deploys viral miRNAs, EBV-miR-BART11 and EBV-miR-BART17-3p, to respectively inhibit the transcriptional repressors FOXP1 and PBRM1. Loss of these checkpoints derepresses the PD-L1 enhancer, producing marked PD-L1 overexpression in EBV-positive GC (19). Collectively, these pathogen-orchestrated mechanisms convert the gastric epithelium into an immunologically privileged niche that accelerates oncogenesis.In response to the aforementioned immune escape mechanisms, current immunotherapeutic approaches for GC primarily encompass ICIs, ACT, mAbs, ADCs, cancer vaccines, and oncolytic viruses, along with other emerging modalities. While immunotherapy demonstrates a more limited response rate compared to conventional treatments, it has exhibited durable clinical responses across diverse cancer types, including GC, with favorable efficacy and manageable toxicity profiles (17). These advantages have established immunotherapy as an increasingly prominent therapeutic strategy for advanced GC.

## 3 Application of immunotherapy for GC

In the clinical management of GC, the integration of immunotherapy follows a structured treatment sequence largely defined by disease stage, biomarker status (e.g., HER2, PD-L1, MSI), and prior lines of therapy. Currently, for patients with advanced or metastatic GC refractory to standard chemotherapy, immunotherapy serves as a critical backbone in the third-line setting and beyond. Notably, nivolumab and pembrolizumab are established options for later-line treatment regardless of PD-L1 status or for PD-L1 positive tumors, respectively. Moving to the second-line setting, strategies are evolving to combine anti-angiogenic agents with chemotherapy, with ongoing research investigating the additive value of ICIs. The most significant paradigm shift has occurred in the first-line treatment of HER2-negative advanced GC, where the combination of PD-1 inhibitors (nivolumab or pembrolizumab) with chemotherapy and trastuzumab (if HER2-positive) has become the standard of care, offering unprecedented survival benefits. Additionally, for the specific subset of HER2-positive patients failing trastuzumab, novel ADCs like trastuzumab deruxtecan (T-DXd) and RC48 have redefined therapeutic options. Beyond these approved standards, emerging modalities such as CAR-T cells targeting Claudin18.2 and cancer vaccines are rapidly progressing through clinical trials, holding the promise to fill existing gaps in refractory disease or to move into earlier lines of therapy. This section will detail the scientific principles and clinical applications of these immunotherapeutic strategies within this hierarchical framework.

### 3.1 ICIs

The theoretical basis of ICIs was established in 1982 by the James Allison team, who identified tumor-specific antigens in a mouse model of T-cell lymphoma using monoclonal antibodies (20). Eventually, in 1995, the Allison team clarified that CTLA-4 is a negative regulatory factor for T-cell activation (21). These mechanistic insights led to the development of the anti-CTLA-4 antibody ipilimumab, which was approved by the FDA in 2011 and became the first immune checkpoint inhibitor to enter clinical application (22). In the field of gastric cancer, the clinical application of immune checkpoint inhibitors achieved a milestone in 2017 when nivolumab was approved by the Japanese regulatory authorities based on the III-stage ATTRACTION-2 trial as a third-line treatment drug (23). ICIs represent a cornerstone of immunotherapy for GC, primarily targeting three key pathways: the PD-1/PD-L1 axis and CTLA-4. Currently approved or investigaticonal ICIs include: (1) anti-PD-1 antibodies (e.g., nivolumab, pembrolizumab, sintilimab, tislelizumab, and tebotelimab), which modulate T-cell activity; (2) anti-PD-L1 antibodies (e.g., durvalumab, atezolizumab, and avelumab), expressed on tumor cells and APCs such as dendritic cell (DC); (3) anti-CTLA-4 antibodies (e.g., ipilimumab), which enhance T-cell priming and activation (24). This section will systematically review the clinical applications of these well-established ICIs while also highlighting novel agents under development.

#### 3.1.1 Targeting the PD-1/PD-L1 axis

The PD-1, primarily expressed on activated T cells, is a pivotal immune checkpoint receptor that modulates immune tolerance. PD-1 was first identified by Ishida and Honjo in 1992 (25), with its ligand PD-L1 (B7-H1) characterized by Dong et al. in 1999 (26). PD-L1, a member of the B7-CD28 immunoglobulin superfamily, functions as a type I transmembrane protein and is widely expressed on immune cells (e.g., macrophages, DC) and various tumor cells, including those in GC, esophageal cancer, and colorectal cancer (27). The binding of PD-1 to PD-L1 initiates inhibitory signaling cascades that impair T-cell proliferation, cytokine secretion, and cytotoxic activity, culminating in T-cell exhaustion and tumor immune evasion (28). Studies indicate that approximately 50% of GC cells express PD-L1, particularly in cases with deep tumor invasion and lymph node metastasis (29). Overexpression of PD-L1 enables GC cells to evade immune surveillance and promotes disease progression by suppressing anti-tumor immune responses (28). The mAbs targeting the PD-1/PD-L1 axis disrupt this interaction, reactivating T-cell-mediated antitumor immunity. These agents have emerged as a transformative therapeutic approach for advanced GC, particularly in biomarker-selected populations.

Many clinical trials have shown that anti-PD-1 treatment has certain efficacy for GC patients (30). For example, the III phase clinical trial on the impact of advanced GC showed that compared with placebo, anti-PD-1 inhibitor nivolumab significantly improved the survival rate of GC patients or patients with gastroesophageal junction cancer (23), and in combination with fluorouracil and platinum as the first-line drug, it could also significantly improve the overall survival (OS) of patients with advanced HER2-negative GC, gastroesophageal junction cancer or esophageal adenocarcinoma (31). The phase III CheckMate-649 trial established nivolumab plus chemotherapy as a standard first-line therapy for advanced HER2-negative GC in 2021 (31). In the II phase clinical trial KEYNOTE-059, researchers found that PD-1 inhibitor pembrolizumab also had similar efficacy and safety for advanced GC patients (32). Pembrolizumab received accelerated approval for PD-L1-positive advanced gastric cancer in 2017 (29).

Beyond these clinically approved ICIs, preclinical investigations have identified multiple novel strategies to enhance the efficacy of PD-1/PD-L1 blockade in GC, particularly through immune microenvironment modulation. Accumulating evidence highlights three promising avenues: targeting co-inhibitory receptors, metabolic reprogramming, and complement system interference. Regarding co-inhibitory receptors, Li et al. reported that co-expression of PD-1 and NKG2A on CD8^+^ T cells synergistically suppresses T cell proliferation and effector functions, suggesting combined blockade as a therapeutic strategy (33). Similarly, Cao et al. demonstrated that V-domain immunoglobulin suppressor of T cell activation (VISTA) blockade reprograms TAMs and exhibits synergistic effects with PD-1 inhibition in GC, underscoring the therapeutic potential of dual VISTA/PD-1 blockade (34). In the realm of metabolic reprogramming, lipid metabolism plays a crucial role in immune escape. Zhang et al. showed that major promiscuous subfamily domain containing 2A (MFSD2A), a lipid metabolism-related protein (35), inhibits COX2-prostaglandin synthesis and suppresses the production of TGFβ1 in GC cells, thereby promoting the activation of CD8^+^ T cells in TME and enhancing the efficacy of anti-PD-1 immunotherapy in GC patients (36). Furthermore, the complement system has emerged as a promising target for improving ICIs’ responses. Zhang et al. revealed that combining anti-PD-1 with C5aR1 blockade synergistically polarizes TAMs toward an inflammatory phenotype, activates CD8^+^T cells, and induces tumor cell apoptosis (37). These findings collectively suggest that targeting co-inhibitory receptors, metabolic pathways, or complement components may potentiate PD-1 blockade in GC.

Beyond combination strategies, recent studies have also uncovered novel intrinsic resistance mechanisms within the PD-1/PD-L1 axis. Liu et al. revealed that Dectin-1+ TAMs exhibit hyperactivation of the PI3K-AKT-mTOR pathway, which may contribute to PD-1 immunotherapy resistance in GC. Mechanistically, a Dectin-1 monoclonal antibody suppressed TAMs immunosuppressive function and restored T cell-mediated cytotoxicity, suggesting that dual Dectin-1/PD-1 targeting could enhance antitumor efficacy (38). Meanwhile, distinct from PD-1 inhibitors that block receptor-ligand interaction to restore T cell activity, PD-L1 inhibitors directly target PD-L1 on tumor cells. For instance, the anti-PD-L1 antibody avelumab demonstrated good tolerability and significant overall response and survival benefits in a phase III trial of advanced GC patients, with enhanced efficacy when combined with other treatments (39). Beyond mAbs, emerging strategies to potentiate PD-1/PD-L1 blockade encompass a wide spectrum of approaches—including natural product-derived small molecules, endogenous hormone-based interventions, RNA interference, and metabolic pathway targeting—as exemplified by the following preclinical findings (**Table 1**).

**Table 1:** New molecular targets for PD-L1-based therapy of GC.

Molecule	Class/category	Mechanism of action	Key preclinical/clinical findings
Avelumab (39)	Antibody drug	Bind to PD-L1 to block the signal and mediate the ADCC effect	Phase III Study on advanced GC has confirmed the tolerability of avelumab; the objective response rates (ORR) and survival in the Japanese subgroup have both improved; The efficacy of the combination therapy has also been enhanced.
Gypenoside (41)	Natural compound	Inhibit PI3K/AKT/mTOR pathway and suppress PD-L1 expression	(a) By binding to STAT3 and inhibiting the expression of p-STAT3 (Tyr705), it suppresses the expression of PD-L1, thereby blocking the transcription of PD-L1. (b) *In vivo*, it inhibits tumor growth, reduces PD-L1 expression, and promotes cell apoptosis.
hsa_circ_0136666 (44)	RNAi	Interact with miR-375 to promote the expression of PRKDC and thereby enhance the stability of PD-L1	(a) Acts as a “sponge” for miR-375, increasing the expression of PRKDC/DNA-PK, phosphorylating PD-L1, enhancing its stability and immune evasion ability. (b) siRNA targeting MLT encapsulated in lipid nanoparticles (NPs) can enhance the efficacy of anti-PD-L1 treatment.
GPX4 (48)	Inducer of ferroptosis	GPX4 knockout induces lipid peroxidation, inhibits ferroptosis and infiltration of TME cells, and downregulates PD-L1 expression	(a) For low-grade tumors, the function of GPX4 is inhibited by regulating histone deacetylase (HDAC) to enhance immunogenicity and reduce the transcriptional level of PD-L1. (b) Knock out GPX4, and then combine it with a PD-L1 blocker. Compared with single therapy, the tumor suppression effect is stronger.

Previous studies have shown that gypenoside has anti-tumor activity in various cancers, including lung cancer, pancreatic cancer, breast cancer, hepatocellular carcinoma, and colorectal cancer (40). Wu et al. demonstrated that gypenoside induces apoptosis of GC cells by inhibiting the PI3K/AKT/mTOR pathway and simultaneously inhibits the expression of PD-L1 in GC cells, thereby enhancing the anti-tumor immunity of T cells (41). These findings provide a compelling molecular rationale for exploring gypenoside as a novel therapeutic adjuvant to enhance the efficacy of immune checkpoint blockade in GC treatment.

Melatonin (MLT), an endogenous indoleamine hormone, is synthesized by various cell types and plays a pivotal role in regulating circadian rhythms. Emerging evidence suggests that MLT also exhibits potent anti-tumor properties (42). Notably, in preclinical studies, Wang et al. demonstrated that exosomes derived from MLT-treated GC cells significantly downregulated PD-L1 expression in macrophages while promoting the secretion of anti-tumor cytokines. This may be the result of the downregulation of PD-L1 in cancer cell-derived exosomes and the upregulation of microRNAs that inhibit PD-L1 expression (43).

Additionally, Miao et al. confirmed that hsa_circ_0136666 is highly expressed in GC and interacts with miR-375 in a sponge-like manner, thereby promoting the expression of protein kinase PRKDC. PRKDC can phosphorylate PD-L1 and enhance its stability, thereby promoting immune escape. Moreover, they found that LNP-small interfering RNA (siRNA) significantly inhibited MDSCs and effectively improved the efficacy of anti-PD-L1 drugs and significantly hindered the recruitment of immunosuppressive cells, thereby inhibiting immune escape. These results highlight the potential of RNA interference (RNAi)-based strategies as a promising therapeutic approach for GC and offer novel insights into PD-L1-targeted therapy (44). Retinoic acid-induced 2 (RAI2) is an innovative tumor suppressor factor (45). In preclinical studies, Lou et al. found that RAI2 can reduce the proliferation and invasion of GC cells by decreasing PD-L1 expression and is associated with TIL, suggesting that it may be an innovative treatment option for GC patients (46).

GPX4 is a key protein for ferroptosis, which can induce lipid peroxidation and inhibit ferroptosis and the infiltration of TME cells (47). Lin et al. demonstrated that GPX4 knockdown in GC cells led to the activation of multiple metabolic and immune-related signaling pathways. Notably, this intervention upregulated the expression of co-stimulatory molecules while significantly reducing PD-L1 messenger RNA (mRNA) levels, suggesting that GPX4 silencing may enhance tumor immunogenicity and counteract immune evasion mechanisms in GC. Consistent with these findings, both *in vitro* and *in vivo* experiments revealed that GPX4 downregulation markedly increased CD8^+^ T cell infiltration and cytotoxic activity. Importantly, their study highlighted the synergistic potential of combining GPX4 suppression with PD-L1 blockade, which substantially improved the efficacy of immunotherapy in GC models (48).

#### 3.1.2 CTLA-4 inhibitors

CTLA-4 is an inhibitory receptor on the surface of activated T lymphocytes and is also the first immune checkpoint described (49). CTLA-4 was first identified by Brunet et al. in 1987 (50), with its critical role as a negative regulator of T-cell activation established by Krummel, and Allison in 1996 (51), work that later contributed to the 2018 Nobel Prize in Physiology or Medicine (52). It can regulate the co-stimulatory signals within the immune synapse between T cells and APCs, thereby negatively regulating the activation of T cells (53). CTLA-4 mainly exerts its inhibitory effect by competing with CD28 for binding to the B7 ligands CD80/CD86 on APCs (27, 54).

Ipilimumab (a fully human IgG1κ antibody) and temelimumab (an IgG2 antibody) are two mAbs targeting CTLA-4. In clinical trials, both have shown limited efficacy in monotherapy for GC, and no significant improvement in progression-free survival (PFS) has been observed (27, 55). Preclinical evidence suggests that dual blockade of CTLA-4 and PD-1 enhances anti-tumor immunity (56). In a phase I/II clinical trial, the ORR of patients with metastatic GC receiving ipilimumab and nivolumab combination therapy is higher than that of patients receiving only nivolumab (57). The combination therapy of these two drugs has been approved for the treatment of advanced GC. Therefore, the combination treatment of CTLA-4 inhibitors with other GC immunotherapies has considerable research value, but the effectiveness of its independent treatment of advanced GC requires further study (58).

### 3.2 Adoptive cell therapy: redirecting immune effectors

Although ICIs have shown significant efficacy in advanced GC, the response rate is still limited by tumor heterogeneity, such as “cold tumors” that do not benefit much from ICI therapy. This section will explore how to reshape the TME through ACT to break through the existing bottleneck.

The development of ACT has a long history, beginning with the pioneering work of Rosenberg and his colleagues at the National Cancer Institute in the United States. They first described the lymphokine-activated killer cell (LAK) therapy in the early 1980s (59), and proved the therapeutic potential of TILs in metastatic melanoma in 1988 (60). The cytokine-induced killer (CIK) cell therapy was later introduced by Wolf et al. in 1991 (61). These foundational studies paved the way for more advanced ACT methods, ACT is an autologous activated lymphocyte therapy (62), mainly including TIL therapy, TCR gene-modified T cells, NK cell therapy, and CAR-T cell therapy etc. (63). ACT enhances the anti-tumor response by transferring lymphocytes and other immune cells, generating effector T cells that specifically target tumor antigens, and enhancing the function of Tregs, ultimately improving prognosis (64). Studies have found that based on the ability of immune cells to recognize and kill tumor cells, ACT can stimulate and restore anti-tumor immunity (65).

Clinically, ACT represents a rapidly evolving class of personalized immunotherapy. While standard ACTs such as CAR-T cells have gained regulatory approval in hematological malignancies, their application in GC is largely investigational and primarily being evaluated in clinical trials for patients with advanced, refractory disease who have exhausted standard treatment options (i.e., third-line or later). ACT has emerged as a promising therapeutic strategy for GC and other malignancies, particularly in cases refractory to conventional treatments (66, 67). Notably, Park et al. demonstrated that ACT generates tumor antigen-specific effector T cells capable of eliciting durable anti-tumor immunity (55). Clinical evidence further supports the efficacy of ACT in advanced GC. In a clinical trial, Takimoto et al. reported that the median survival time of advanced GC patients was significantly prolonged to 31.4 months and 32.6 months in cohorts receiving ACT combined with chemotherapy or surgery, respectively, compared to 15.1 months in the chemotherapy-alone group (62). Additionally, a systematic review and meta-analysis of clinical trials by Shen et al. revealed that ACT combined with adjuvant therapy significantly enhanced OS and PFS in GC patients (68). Collectively, these findings underscore the potential of ACT as a beneficial therapeutic approach for advanced GC.

#### 3.2.1 CAR-T cell therapy

CAR-T has been proven to be a promising GC treatment strategy (69). CAR-T cell therapy involves genetically modifying T cells to express engineered receptors, which are specifically designed to recognize and target the specific antigens present in cancer cells (70–72). This genetic alteration activates T cells, enabling them to mobilize the immune system to recognize and eliminate tumor cells (70–73). CAR-T cells are categorized into four distinct generations based on their intracellular signaling domain architecture (71). The foundational first-generation CARs, pioneered by Gross et al. in 1989, were constructed by fusing single-chain variable fragments of mAbs with immune receptor tyrosine-based activation motifs (ITAMs) such as CD3ζ or FcϵRIγ (74, 75). To address limitations in cell expansion and cytokine production observed in first-generation constructs, Finney et al. developed second-generation CARs incorporating a single co-stimulatory domain (76). Subsequent advances led to third-generation CARs featuring two tandem co-stimulatory molecules, which significantly enhance T-cell effector functions and persistence *in vivo* (77). The fourth-generation CAR-T cells can secrete a large amount of cytokines to the tumor site to activate innate immune responses and enhance anti-tumor effects (78).

Compared with other ACTs, CAR-T cells directly recognize tumor surface-related antigens, independent of MHC restriction (79). Claudins (CLDN) are the main membrane protein family that form tight junctions. Claudin18.2 (CLDN18.2) is a member of the CLDN family and is specifically expressed in normal gastric mucosal tissues. Claudin18.2 is also abnormally expressed in various cancers, including GC, esophageal cancer, pancreatic cancer, colorectal cancer, lung cancer, and hepatocellular carcinoma (80, 81). Clinical development status of CAR-T therapies targeting different membrane proteins in GC. Among them, CAR-T cells targeting CLDN18.2 are prepared by transduction with lentiviral vectors have demonstrated the most substantial translational progress, evolving from early proof-of-concept studies to the first global randomized controlled trial in solid tumors (82). Initial clinical validation was provided by a case report from Botta et al., which reported the successful implementation of anti-CLDN18.2-targeted CAR-T cells (CT041) in the last-line metastatic GC and detailed the continuous complete responseof target lesions on clinical, molecular, and imaging levels until 8 months after the first infusion. CT041 showed promising anti-tumor activity even in chemotherapy-refractory patients who failed 4-line systemic treatment and multiple HIPEC cytoreductive surgeries (83). This promising activity prompted rigorous clinical evaluation. Subsequently, Qi et al. reported comprehensive data from a phase 1 trial (NCT03874897) involving 98 patients with gastrointestinal cancers who received satricabtagene autoleucel (satri-cel). The 2024 final analysis revealed an ORR of 38.8% and a disease control rate (DCR) of 91.8%, with a median PFS of 4.4 months and a median OS of 8.8 months (84). Marking a pivotal milestone in the development of cellular immunotherapy for solid tumors, a randomized, open-label, phase II trial (NCT04581473) was conducted in 2025. This study demonstrated a significant survival benefit in the intent-to-treat population, with satri-cel achieving a median PFS of 3.25 months (95% CI 2.86–4.53) compared to 1.77 months (95% CI 1.61–2.04) in the treatment physician’s choice (TPC) group (risk ratio 0.37 [95% CI 0.24-0.56]; one-sided log-rank p < 0.0001). While safety profiles indicated frequent grade 3 hematological toxicities and cytokine release syndrome (95%), the manageable risk-benefit ratio established satri-cel as a novel and effective third-line treatment option for advanced GC (85).

In addition to targeting CLDN18.2, CAR-T cells also act by targeting mesothelin (MSLN), NK receptor group 2 (NKG2D), HER2 and mucin 1, thereby providing more immunotherapy avenues for GC. MSLN is a membrane protein found to be highly up-regulated in GC cells (71). MSLN-specific CAR-T cells have been successfully used in GC (86). Currently, MSLN−specific CAR−T cells remain at the preclinical stage for GC. The results of Zhang et al. showed that the third-generation MSLN-CAR-T cells could significantly increase the levels of T cells and cytokines, thereby weakening the growth of MSLN-positive GC cells (87). This indicates that MSLN-CAR-T cells are promising candidates for GC treatment. In addition, Zhao et al. also found that human hyaluronidase PH20 could further enhance the anti-tumor activity of MSLN-CAR-T cells against GC (88). Another compelling target is NKG2D, a homodimeric C-type lectin-like receptor expressed on cytotoxic T cells and NK cells (89). Preclinical studies by Tao et al. demonstrated robust *in vitro* and *in vivo* anti-tumor activity of NKG2D-CAR-T cells in GC models. Their findings revealed that NKG2D-CAR-T cells significantly enhanced tumor cell lysis and inhibited the progression of established GC xenografts. Notably, cisplatin was found to upregulate the expression of NKG2D ligands on GC cells, thereby sensitizing them to NKG2D-CAR-T cell-mediated cytotoxicity (90). These observations highlight the potential of NKG2D-CAR-T cells as a viable therapeutic option for GC, particularly in combination with conventional chemotherapy, but they have not yet entered clinical trials for GC.

Furthermore, Preclinical studies have demonstrated the incorporation of PD1/CD28 co-stimulatory receptors into CAR-T cells enhances their therapeutic efficacy (91). This is mainly achieved by blocking the T-cell activity inhibition caused by the binding of PD-1 and PD-L1 and activating T-cell proliferation and differentiation through CD28 (92). Additionally, this modification promotes the secretion of immunostimulatory cytokines, including IFN-γ and TNF-α, which contribute to robust anti-tumor immune responses (93, 94). Recently, Zhang et al. found that in GC cells, kynurenic acid (KynA) produced by interleukin-4-induced-1 (IL4I1) on tryptophan can significantly activate Aryl hydrocarbon receptor (AHR) Nuclear translocation of CAR-T cells drives CD8^+^ CAR-T cell exhaustion. In addition, collagen in the extracellular matrix can further aggravate GC immune escape by activating IL4I1-AHR signaling (95). Therefore, targeting this pathway may enhance the efficacy of CAR-T cell therapy in GC, but this strategy remains strictly preclinical.

#### 3.2.2 TIL therapy

TILs represent a critical cellular component within the TME, typically comprising diverse T cell clones that collectively reflect the host’s endogenous anti-tumor immune response (96). These lymphocytes exhibit the unique capability to recognize and target tumors through multiple TAAs presented by various HLA molecules (97). Importantly, the utilization of autologous TILs enhances tumor antigen recognition while simultaneously improving treatment specificity and reducing off-target toxicity (72). Accumulating clinical evidence has demonstrated the pivotal role of TILs in suppressing tumor progression across multiple cancer types, including ovarian, breast, and colorectal carcinomas (96, 98, 99). Furthermore, the abundance and functional status of TILs have been consistently correlated with the clinical outcomes of active cancer immunotherapies.

TIL therapy was first tested in a mouse model in 1985, and then in 1988, it was transformed into the first clinical trial for patients with metastatic melanoma (100). In 2024, Lifileucel became the first FDA-approved TIL product and officially entered clinical practice (101). However, no TIL product has yet been approved for GC. Several studies have investigated the prognostic value of TILs in GC patients, which represents clinical observation rather than therapeutic intervention. For instance, Kang et al. conducted a related analysis on 120 patients with EBV-related GC and found that high TIL was independently associated with good disease-free survival (DFS) or recurrence free survival (RFS). This association suggests that robust TIL infiltration enhances the host’s cellular immune response, thereby exerting potent anti-tumor effects (102). Emerging evidence from recent studies has demonstrated the potential of TILs and their functional subsets in guiding immunotherapy selection for GC patients (103–106). As highlighted by Saldanha et al., TILs serve as the biological foundation supporting ICIs efficacy while simultaneously informing adoptive immunotherapy strategies for GC (107, 108). In summary, while TILs have strong prognostic value in GC and the TIL therapy platform has achieved regulatory approval in melanoma, the therapeutic application of TIL infusion in GC currently resides at clinical trial stage, with no approved products and no published efficacy results specific to GC. Further trials are needed to determine its clinical utility in this disease setting.

#### 3.2.3 TCR-T therapy

TCR-T therapy represents an advanced form of adoptive cell immunotherapy that utilizes genetic engineering to redirect T cell specificity, emerging as a promising therapeutic strategy for cancer treatment (109, 110). Unlike CAR T-cell therapy, TCR-T cells recognize intracellular tumor antigens presented by MHC molecules, enabling targeted elimination of malignant cells (72). In preclinical studies, Wang et al. demonstrated that TCR diversity and clonal expansion patterns significantly correlate with clinical outcomes in GC patients, particularly through their association with dynamic changes in CD8^+^PD-1+ T cell subsets (111). Despite its therapeutic potential, this approach faces challenges, as the use of high-affinity TCRs may lead to severe adverse effects, including on-target/off-tumor toxicity and cytokine release syndrome (58).

Compared with TIL therapy, engineered TCR-T cells have strong antigen specificity and functional affinity, target patient-specific tumor antigens, and can minimize damage to normal tissues to the greatest extent (97). It is worth noting that engineered TCR-T cells can not only recognize membrane proteins but also recognize intracellular antigens presented by MHC molecules, covering a wider range of tumor neoantigens and therapeutic targets (63). Furthermore, emerging evidence suggests synergistic effects when combining TCR-T cell therapy with ICIs or other modalities, which may potentiate T cell anti-tumor activity and improve clinical outcomes (63, 112).

### 3.3 The mAbs and ADCs

While ICIs and ACTs have revolutionized the management of advanced GC, their efficacy remains constrained by primary resistance mechanisms (e.g., low tumor immunogenicity) and practical challenges such as manufacturing complexity. As a versatile strategy, ADCs that combine the precision of targeted therapy with the potency of cytotoxic payloads can bridge these gaps. By selectively delivering chemotherapeutic agents or immune modulators to antigen-expressing tumor cells, ADCs not only circumvent systemic toxicity but also overcome resistance to conventional immunotherapies. The clinical application of ADCs is rooted in decades of technological accumulation. This therapeutic concept can be traced back to the 1960s, and its core idea is to use antibodies to selectively deliver cytotoxic payloads to tumor cells. After long-term technological iterations, gemtuzumab ozogamicin was approved by the FDA in 2000 for the treatment of acute myeloid leukemia, marking the official entry of ADCs drugs into clinical practice (113). Recent breakthroughs, such as T-DXd achieving unprecedented responses in HER2-low GC in a Phase II Trial, underscore their potential to redefine treatment paradigms for biologically distinct subgroups (114). We have summarized the clinical research on GC immunotherapy related to these monoclonal antibody drugs in **Table 2**, which helps us better understand the current research progress. In the clinical therapy for GC, mAbs and ADCs are utilized across various lines of therapy. Trastuzumab remains the standard first-line treatment for HER2-positive GC. For patients progressing on trastuzumab, HER2-targeted ADCs such as T-DXd and RC48 have become preferred options in the second-line or third-line settings. Meanwhile, anti-angiogenic mAbs like ramucirumab are established in the second-line setting, either as monotherapy or combined with paclitaxel.

**Table 2:** Key GC immunotherapy clinical trials.

Trial name	Phase	Patient population	Intervention	Control	Primary endpoint results	Key safety findings
FORTITUDE-102 (130)	III	FGFR2b-overexpressing GC/GEJ (1L)	Bemarituzumab + Chemo + Nivolumab	Chemo + Placebo	Pending	Pending
*In vitro* combination study (135)	preclinical	Five human GC cell lines: (a) MET positive: Hs746T, MKN45, SNU620; (b) MET negative: AGS, SNU638	(a) Single/double drug treatment for 48 hours: Tepotinib, Paclitaxel, RAM (b) Combination: Tepotinib + Paclitaxel vs. RAM + Paclitaxel	Dual-drug combination self-control: tepotinib + paclitaxel vs. RAM + paclitaxel	Tepotinib combined with paclitaxel is significantly superior to RAM combined with paclitaxel.	Not evaluated
INTEGA trial (143)	II	ERBB2 (HER2)-positive advanced gastroesophageal adenocarcinoma (EGA)	Trastuzumab + Nivolumab + mFOLFOX6	Trastuzumab + Nivolumab + Ipilimumab arm	OS rate 70% (at 12 months), median PFS 10.7 months	Safety: 88% with grade 3 adverse events, 23% of leukopenia and 11% of neuropathy
NCT04908566 (144)	II	Locally advanced gastric/gastric-cardiac junction adenocarcinoma	PD-1 inhibitors	None	Main pathological response (MPR, ≤10% viable tumor cells): MPR rate: 53.3%	Unreported grade ≥ 3 treatment-related adverse events (TRAEs)
CheckMat e 649 (145)	III	Late-stage GC/GEJC/EAC patients in China	Nivolumab + Chemotherapy:	Simple chemotherapy	Median OS (14.3 months vs 10.2 months; HR 0.61 [95% CI: 0.44 - 0.85]), median PFS (8.3 vs 5.6 months; HR 0.57 [95% CI: 0.40 - 0.80]), ORR (66% vs 45%), and median DOR (12.2 vs 5.6 months)	No new safety signals
NCT04609176 (146)	II	AFP-positive advanced G/GEJ adenocarcinoma	Karelixibumab + Apatinib + SOX as first-line treatment, Karelixibumab + Apatinib for maintenance treatment	None	ORR was 66.7% (95% CI: 49.0 - 81.4)	No new safety issues were identified
ChiCTR2100044088 (147)	IB	Late-stage GC/GEJC	β-glucan + Karelixibumab + SOX	None	ORR was 60%	Safety is acceptable
NCT04190745 (148)	II	Late-stage GC/EGJC	Apatinib + Turepipramab	Irinotecan monotherapy, paclitaxel monotherapy, or docetaxel monotherapy	1-year survival rate: 43.3% vs 42.3% (P = 0.903)	≥3 grade TRAE: 24.0% vs 34.6%
NCT05025033 (149)	II	Late-stage GC/GEJ adenocarcinoma	Cindlimibumab + Apatinib + Chemotherapy (Paclitaxel/Ilicotinoin)	None	ORR: 53.6% (95% CI: 33.9 - 72.5) and median PFS: 8.5 months (95% CI, 5.4 - 11.5)	3–4 grade adverse events

#### 3.3.1 Anti-VEGF therapy

Tumor neovascularization hinders the infiltration of immune cells that respond to tumors and promotes an immunosuppressive TME, enabling tumors to resist immunotherapy (115). VEGF can promote neovascularization, impair the interaction between leukocytes and endothelial cells, and inhibit the infiltration of immune cells into the TME by reducing adhesion molecules, and enhance the presence of Tregs and other immunosuppressive cells, thereby regulating the immune response and allowing tumor cells to escape surveillance (28, 116). The VEGF inhibitor ramucirumab (RAM) is a monoclonal antibody that binds to vascular endothelial growth factor receptor (VEGFR)-2, mainly inhibiting the VEGF-A/VEGFR-2-mediated vascularization signal cascade reaction (117). Apatinib is a tyrosine kinase inhibitor (TKI) targeting VEGFR-2 (118). Both RAM and apatinib have been proven effective in preclinical and advanced GC/GEJC patients and have been approved as monotherapy or in combination with paclitaxel (119–122). Mechanistically, studies have shown that RAM targeting VEGFR2 in advanced GC/GEJC patients leads to increased CD8^+^ T cell infiltration and PD-L1 expression in the TME (123). The REGARD trial compared the efficacy of RAM monotherapy with placebo in patients with GC/GEJ adenocarcinoma. The results of this phase III clinical trial showed that the median OS of the RAM group was 5.2 months, while that of the placebo group was 3.8 months (120). The RAINBOW trial compared the efficacy of RAM combined with paclitaxel with placebo combined with paclitaxel in advanced GC patients. This double-blind randomized phase III trial indicated that the median OS of the RAM plus paclitaxel group (9.6 months) was significantly longer than that of the placebo plus paclitaxel group (7.4 months) (27, 119). Angiogenesis inhibitors, including RAM and apatinib, have demonstrated the potential to augment anti-tumor immune responses by modulating immunosuppressive activity. This immunomodulatory effect supports the rationale for combining angiogenesis inhibitors with other immunotherapeutic agents, which may yield synergistic anti-tumor efficacy (117, 118). For instance, a multicohort, non-randomised, open-label, phase 1a/b trial in pretreated patients with advanced GC or gastroesophageal junction adenocarcinoma revealed promising anti-tumor activity with dual blockade of the VEGF-VEGFR2 and PD-1-PD-L1 pathways (e.g., RAM plus pembrolizumab) (28, 124). Further mechanistic insights were provided by Tao et al., who reported that low-dose apatinib may potentiate the efficacy of PD-1 inhibitors by promoting tumor vascular normalization, alleviating intratumoral hypoxia, and reprogramming the immunosuppressive TME, thereby enhancing anti-GC activity (125). Additionally, preclinical emerging evidence suggests a positive correlation between VEGF and CD47 expression in GC, implicating coordinated immune evasion mechanisms. Consequently, combined targeting of VEGF and CD47 has been shown to remodel the TIME and amplify anti-tumor responses, for example, through anti-CD47 antibodies or bispecific fusion proteins (such as SIRPα-VEGFR1) (115).

#### 3.3.2 Anti-FGFR therapy

The fibroblast growth factor (FGF) signaling pathway mediates its biological effects through FGFRs, which play crucial roles in regulating cellular proliferation, differentiation, angiogenesis, immune modulation, and metabolic homeostasis (126). As members of the receptor tyrosine kinase family, FGFRs are transmembrane proteins whose aberrant activation or overexpression has been implicated in the pathogenesis of various malignancies, including GC, lung cancer, and bladder cancer. Currently, several therapeutic strategies targeting the FGF/FGFR axis have been developed, including FGFR-specific mAbs, small-molecule TKIs, DNA/RNA aptamers, and FGF ligand capture (127). Notably, emerging preclinical and clinical evidence suggests that combining FGFR inhibitors, particularly TKIs, with ICIs may represent a promising therapeutic approach for cancer treatment, potentially overcoming resistance to monotherapy (128). Clinically, targeting FGFR remains an investigational strategy in GC, primarily focused on the subset of patients with FGFR2b overexpression. Bemarituzumab is a specific monoclonal antibody against FGFR2, showing promising clinical efficacy in the treatment of GC (129). Currently, a Phase III FORTITUDE-102 study is further evaluating the efficacy of bemarituzumab combined with chemotherapy and nivolumab in treating FGFR2b-overexpressing GC and GEJ (130). Additionally, Popiel et al. are developing a small molecule FGFR1–3 kinase inhibitor, CPL304100, which has shown efficacy in FGFR-dependent cell lines and patient-derived tumor xenograft (PDTX) *in vivo* models. This preclinical result has prompted the initiation of a phase I clinical trial (NCT04149691) to further evaluate CPL304100 as a novel anticancer therapy. It is expected to become a potential candidate drug for patients with FGFR gene aberrations and GC (131).

#### 3.3.3 Anti-c-MET therapy

The c-MET represents a distinct subfamily of receptor tyrosine kinases and serves as the primary receptor for hepatocyte growth factor (HGF) (132). Accumulating evidence demonstrates that aberrant activation of the HGF/c-MET signaling pathway plays a pivotal role in gastric carcinogenesis, tumor progression, metastatic dissemination, and poor clinical outcomes (133). Given its critical involvement in GC pathogenesis, targeted inhibition of this pathway has emerged as a promising therapeutic strategy.

Despite the biological rationale, clinical development of c-MET inhibitors in GC has faced challenges, and no agents are currently approved specifically for this indication. Tepotinib, a highly selective c-MET inhibitor, has demonstrated promising therapeutic potential in GC (134). For instance, the *in vitro* findings of Sun et al. indicate that tepotinib combined with paclitaxel exhibits superior efficacy in suppressing tumor growth and metastasis compared to RAM plus paclitaxel, particularly in GC cases harboring MET exon 14 skipping mutations or phosphorylated MET overexpression. Further clinical studies are required to confirm the therapeutic effects of these regimens (135). Beyond small-molecule inhibitors, several mAbs targeting the c-MET pathway are currently under evaluation in Phase II/III randomized clinical trials for advanced GC (135), including onartuzumab (136–138), rilotumab (137, 139), and emibetuzumab (140, 141). Among them, onartuzumab can inhibit the dimerization of the c-MET receptor, block the downstream HGF/c-MET signaling pathway, and exert anti-GC effects (142). An I-phase clinical trial result showed that onartuzumab treatment led to a sustained complete response (> 2 years) in a chemotherapy-refractory GC patient with liver metastasis at pathological stage PT3N1M1 (133). However, the clinical development of c-MET-targeted agents has faced challenges, likely attributable to the intricate molecular mechanisms underlying HGF/c-MET signaling and tumor heterogeneity (73). These findings underscore the need for further research to optimize patient selection strategies and therapeutic regimens in c-MET-driven GC.

#### 3.3.4 ADCs

ADCs represent a novel class of targeted anti-tumor agents that combine high therapeutic efficacy with precise tumor selectivity, offering a promising therapeutic strategy for GC and other malignancies (150, 151). These innovative molecules consist of three key components: a monoclonal antibody that specifically targets TAAs, a potent cytotoxic payload, and a stable linker connecting these two moieties. The unique design of ADCs enables selective delivery of cytotoxic drugs to tumor cells while minimizing systemic toxicity, resulting in superior therapeutic effects compared to naked mAbs (152, 153). This targeted approach significantly enhances the therapeutic index by concentrating the cytotoxic payload within tumor tissues, thereby overcoming some limitations of conventional chemotherapy and monoclonal antibody therapy.

In the clinical management of HER2-positive GC, ADCs have become integral, particularly in the second-line and later settings. T-DXd is a novel HER2-targeting ADC that demonstrates multifaceted immunomodulatory effects in HER2-positive (HER2+) GC. Beyond its direct cytotoxic payload delivery, T-DXd enhances antitumor immunity by facilitating HER2 uptake by DC, promoting cytotoxic T lymphocyte activation, and upregulating PD-L1 expression (73). Building on its approval for third-line therapy, clinical evidence from a randomized phase II trial revealed superior 12-month OS rates in treatment-naïve HER2+ GC patients receiving T-DXd combined with nivolumab and ipilimumab compared to those treated with T-DXd plus FOLFOX chemotherapy, suggesting a potential role for T-DXd in first-line combination strategies (154). Mechanistically, preclinical studies have demonstrated that co-administration of T-DXd with pyrotinib, a HER2 TKI, synergistically enhances ADC endocytosis, leading to improved antitumor efficacy while mitigating treatment-related adverse effects (143). Notably, ARX788, an alternative anti-HER2 ADC, has exhibited superior activity to T-DXd in xenograft models of GC with low HER2 expression, suggesting its potential clinical utility in this challenging patient subset (114).

Disitamab vedotin (RC48), a novel anti-HER2 ADC, has demonstrated significant anti-tumor efficacy in GC patients, including those with low HER2 expression (153). An I-phase study highlighted its robust clinical activity and acceptable safety profile in advanced HER2+ GC (155). These findings were further corroborated by an open-label, multicenter, phase II trial, which reported promising clinical responses and survival benefits in patients with locally advanced or metastatic HER2-overexpressing GC receiving RC48 as a later-line therapy (156).

Notably, emerging evidence suggests that combining RC48 with ICIs yields superior outcomes compared to RC48 monotherapy. This combination has been associated with enhanced ORR, disease control rates (DCR), and median PFS, likely attributable to the ability of ADCs to upregulate PD-L1 and MHC-I expression in tumor cells, thereby sensitizing them to immunotherapy (157–159). For instance, a recent study demonstrated that RC48 plus ICIs exhibited remarkable efficacy and manageable toxicity in HER2+ or HER2-low-expressing advanced/metastatic GC patients undergoing third-line or later treatment, further supporting the synergistic potential of this therapeutic strategy (159). Moreover, Cai et al. also developed DX126-262, a new type of anti-HER2 ADC. Currently, the Phase II clinical trial results show that the monotherapy with DX126–262 has demonstrated good efficacy in HER2+ advanced GC patients. Additionally, preliminary results suggest that combining DX126–262 with 5-fluorouracil (5-FU) and cisplatin may represent a viable therapeutic strategy, warranting further investigation (151). These advances in ADC development offer valuable insights for optimizing treatment regimens in HER2+ GC, potentially improving clinical outcomes for this patient population.

### 3.4 Cancer vaccine

Despite passive immunotherapy has made remarkable advances, including ICIs, ACTs, ADCs, these approaches often face limitations in sustaining durable immune memory or addressing antigen-negative recurrences. In this context, cancer vaccines have re-emerged as a proactive strategy to prime *de novo* anti-tumor immunity by eliciting long-lasting T cell memory against TAAs or neoantigens. The first engineered therapeutic cancer vaccine, Melacine, was developed in 1998 for melanoma, marking a pivotal milestone in cancer immunotherapy history (160). Cancer vaccine is an immunotherapy targeting antigens expressed exclusively by cancer cells (161), aiming to overcome tumor-induced immunosuppression, enhance immunogenicity, activate the patient’s immune system, and trigger humoral immunity and cellular immunity (73, 162, 163).

Recent innovations in vaccine platforms (e.g., mRNA-LNP) and combinatorial regimens (e.g., vaccine + PD-1 blockade) are overcoming historical barriers of low immunogenicity, positioning vaccines not merely as standalone therapies but as immune architects capable of converting ‘cold’ tumors into immunologically responsive ecosystems. The existing cancer vaccines mainly include cell vaccines, protein/peptide vaccines, and gene (DNA, RNA, and virus) vaccines (28). Notably, cancer vaccines exhibit distinct advantages over other immunotherapeutic strategies, offering high antigen specificity, favorable safety profiles, and generally excellent tolerability in clinical settings (153). Currently, cancer vaccines for GC are primarily in the experimental stage, with most clinical trials focusing on their use as adjuvant therapy to prevent recurrence after surgery or in combination with ICIs for advanced disease. No cancer vaccine has yet been approved as a standard monotherapy for GC.

#### 3.4.1 DC vaccines

DC vaccines are prepared by expressing specific TAAs on the surface of patients’ DCs, followed by treatment with immune-stimulating adjuvants and then administered to patients autologously (161). In 2002, Kono et al. described a Phase-1 vaccination trial in GC patients and confirmed the feasibility and effectiveness of DC vaccines pulsed with HER-2-derived peptides (164).

#### 3.4.2 Protein/peptide vaccines

Protein/peptide vaccines mainly target TAAs and have been used to stimulate specific lymphocyte responses in GC. Based on TAAs HER2/neu-derived peptides and melanoma-associated antigen, these peptides can induce cytotoxic T cells to fight against tumors (165). Due to the poor immunogenicity of TAAs, the immune effect of protein/peptide vaccines can be enhanced by combining with inflammatory adjuvants or other immunomodulators (161). Previous studies evaluated the anti-GC activity of peptide vaccines such as G17DT, OTSGC-A24, and VEGFR. Among them, G17DT is a vaccine that can counteract gastrin-17. G17DT has good efficacy and tolerance in the treatment of advanced cancer (27). Another study showed that patients with untreated metastatic GC or gastroesophageal adenocarcinoma may exhibit a higher median OS rate after vaccination with G17DT in a multicenter, phase II study (30, 166).

#### 3.4.3 Gene vaccines

Gene vaccines represent a promising immunotherapeutic approach, encompassing DNA vaccines, RNA vaccines, and viral vector-based vaccines. These vaccines function by delivering genetic material encoding TAAs or antigenic epitopes into APCs, thereby stimulating specific immune responses against cancer cells (161). DNA vaccines typically employ plasmid vectors or viral vectors to facilitate the uptake of antigen-encoding DNA sequences by APCs. Following cellular internalization, the DNA is transcribed and translated into protein antigens, which are subsequently processed and presented via MHC molecules. This process ultimately activates both humoral and cellular immune responses (167). In contrast, RNA vaccines primarily utilize mRNA molecules that carry the genetic information of target antigens. Upon delivery into host cells, the mRNA is rapidly translated into antigenic proteins, eliciting robust and broad immune activation against tumor cells (168). Viral vector-based vaccines commonly exploit modified vaccinia viruses, adenoviruses, or herpes simplex virus type 1 (HSV-1) as delivery vehicles. Among these, vaccinia virus-based platforms offer distinct advantages, particularly their large transgene capacity that enables incorporation of multiple or sizable antigen-encoding sequences (161).

### 3.5 Emerging strategies to enhance immunotherapy efficacy

Although ICIs, ACT, ADCs, and cancer vaccines have markedly expanded the therapeutic armamentarium for GC, their clinical reach is curtailed by inter- and intra-tumoral heterogeneity, TIME niches, and dose-limiting off-target toxicities. To overcome these challenges, emerging multidisciplinary strategies are being developed, integrating precision engineering and systems biology approaches to enhance therapeutic outcomes. Nano-delivery systems now offer spatiotemporal command over immunomodulators, amplifying intra-tumoral drug exposure while curtailing systemic toxicity. Simultaneously, physical-energy triggers induce immunogenic cell death and remodel the stromal barrier, synergizing with ICIs or ACT. Integration of immune adjuvants, phytochemicals and microbiota-derived exosomes further deciphers and rewires the immunosuppressive TIME, enabling rational, patient-specific combination regimens that transcend the limitations of conventional monotherapies. It is important to note that most of these novel approaches are currently in the preclinical stage of development.

#### 3.5.1 Precise regulation of nano-delivery systems

Nanotechnology has forged a new therapeutic paradigm in GC, wherein engineered NPs function as multifunctional vectors that integrate targeted drug delivery with multimodal imaging. The conceptual foundation of nanomedicine in oncology traces to the 1990s, with the first nano-drug Doxil gaining regulatory approval in 1995 (169). These nanoscale constructs surmount the pharmacokinetic and biodistribution constraints of conventional agents by enhancing bioavailability, achieving tumor-selective accumulation, and improving systemic biocompatibility. A paradigmatic illustration is the hypoxia-activated TH-302 NP platform developed by Wang et al. In preclinical studies, it was shown that under hypoxic conditions, TH-302 NPs potently inhibit GC cell proliferation and induce apoptosis. Critically, when combined with anti-PD-1 therapy, TH-302 NPs orchestrate a synergistic reprogramming of the TIME: they downregulate PD-L1 and HIF-1α, augment CD8^+^ T-cell tumor infiltration, and amplify effector cytokines (IFN-γ, TNF-α) and cytotoxic mediators (granzyme B). This multimodal immunomodulation converts an immunosuppressive milieu into one permissive for durable anti-tumor immunity (170).

Recent advancements in NPs technology have enabled the targeted delivery of molecular therapeutics designed to counteract intrinsic immune evasion mechanisms in GC. A preclinical seminal study by Li et al. revealed that the circular RNA circRHBDD1 plays a crucial role in tumor immune escape by upregulating PD-L1 expression and suppressing CD8^+^T cell infiltration. To therapeutically exploit this finding, the researchers developed a novel nano-delivery system comprising poly (lactide-co-glycolide)-polyethylene glycol (PLGA-PEG) NPs loaded with siRNA targeting circRHBDD1. This engineered nanoplatform demonstrated efficient *in vivo* silencing of oncogenic circRHBDD1, thereby sensitizing treatment-refractory GC models to anti-PD-1 checkpoint blockade therapy. These findings not only provide compelling mechanistic evidence but also establish a proof-of-concept for combining RNAi with nanotechnology to overcome immunotherapy resistance in GC (171).

#### 3.5.2 Immunological synergistic effect of physical energy therapy

Photodynamic therapy (PDT) and hyperthermia each amplify anti-PD-1 efficacy in GC via distinct yet complementary mechanisms.

The concept of PDT originated in 1900 and was rediscovered several times. However, the clinical era truly began in the early 1970s with the research conducted by Dougherty and his team (172). At the beginning of the 19th century first clinical trials with systemically induced fever were performed on malignant tumors (173). Recent studies have shown that liposome-modified photosensitizer Icy7 (LLI), when combined with immunogenic PDT and neutrophil-targeted immunotherapy, can substantially enhance the efficacy of anti-PD-1 therapy. This combinatorial approach induces robust TME remodeling and systemic anti-tumor immune responses, primarily through three mechanisms: (1) promoting DC maturation, (2) increasing CD8^+^T cell infiltration, (3) reducing M2-like TAM accumulation. Furthermore, this strategy upregulates PD-L1 expression on DCs, which may potentiate the therapeutic effect of ICIs (174).

Similarly, hyperthermia, as another promising physical modality, elevates intra-tumoral temperature to induce thermal ablation, suppress proliferation, invasion and migration, and trigger apoptosis via down-regulation of PLEK2/PD-L1 (175, 176). Collectively, these physical-energy modalities provide mechanistic rationale for combining hyperthermia or PDT with immune-checkpoint inhibitors in GC.

#### 3.5.3 Microenvironment regulation by immunoadjuvants and natural products

Immunoadjuvants represent a promising approach to remodel the TIME, and their synergistic application with ICIs may offer a viable strategy to overcome primary immunotherapy resistance. For instance, nanovaccines engineered from CpG oligodeoxynucleotides (CpG-ODN) and the GC-specific antigen MG7 have demonstrated robust anti-tumor efficacy in preclinical studies, highlighting their therapeutic potential (175).

The combination of traditional Chinese medicine (TCM) and natural products with ICIs demonstrates promising synergistic effects in GC immunotherapy (177). TCM formulations have been used empirically for centuries, though their systematic evaluation in combination with modern immunotherapy is a phenomenon of the past decade (178). Modified Bu-zhong-yiqi Decoction (mBYD) inhibits the progression of GC by increasing the infiltration of tumor lymphocytes, reducing the infiltration of PD-1 and PD-L1, and down-regulating PD-1 in peripheral blood (179). Modified Jianpi Yangzheng Decoction (mJPYZ) modulates the TME by decreasing the secretion of extracellular vesicles (EVs) containing pyruvate kinase M2 (PKM2) from GC cells. This reduction inhibits PKM2-mediated macrophage polarization toward M2-like TAMs, thereby attenuating tumor progression (180). Similarly, Jianche Yangzheng Xiaozhong Decoction (JPYZXZ) suppresses GC progression by targeting extracellular vesicle PD-L1, which effectively reduces MDSCs expansion and reverses immunosuppression in the TME (181). Overall, these data indicate that the adjuvants based on TCM principles can serve as valuable partners in PD-L1 targeted immunotherapy for GC, and have clinical application value.

#### 3.5.4 The intervention potential of microbiota and exosomal vesicles

The gastrointestinal tract harbors a highly diverse microbial community, which has been increasingly linked to enhanced efficacy of ICIs therapy in cancer treatment (28).

Within this context, H. pylori has emerged as a pivotal determinant of immunotherapy outcome since about 2020. Increasing evidence indicates that the presence of Helicobacter pylori can affect the efficacy of PD-1/PD-L1 blockade therapy in gastric cancer (182). For instance, Che et al. reported that advanced GC patients positive for H. pylori exhibited a higher likelihood of non-response to PD-1 antibody therapy, along with significantly shorter PFS and OS, compared to H. pylori-negative patients (183). One plausible mechanism involves H. pylori neutrophil-activating protein (HP-NAP), a virulence factor that modulates immune responses by activating neutrophils and monocytes, promoting cytokine production, enhancing DC maturation, and polarizing macrophages toward an anti-tumor phenotype (184). Beyond microbial modulation, EVs have emerged as another viable target for enhancing immunotherapy efficacy. EVs, including exosomes, were first characterized by Trams et al. in 1981 (185), with their role in cancer immune evasion recognized in the 2005 (186) and their therapeutic exploitation beginning circa 2015 (187). Shen et al. demonstrated that lysine-specific demethylase 1 (LSD1) ablation reduces EV-associated PD-L1 levels, thereby restoring T-cell activity in GC (188). Complementarily, hybrid EVs derived from DCs and induced pluripotent stem cells (iPSCs) have been engineered to co-deliver PD-1 antibodies and doxorubicin, eliciting synergistic chemo-immunotherapy and improving prognosis in pre-clinical GC models (189, 190). These findings position microbiota- and EV-centric strategies as actionable adjuncts to precision immunotherapy in GC.

## 4 Biomarkers: towards precision immunotherapy in GC

Despite the significant improvement in the prognosis of GC patients with the application of ICIs, the clinical outcomes for most advanced-stage cases remain poor, with a 5-year survival rate below 30% (191). The principal obstacle resides in the profound molecular and phenotypic heterogeneity of GC, which yields highly variable responses to immunotherapy. Consequently, reliable predictive biomarkers are urgently required to refine patient selection across disease stages (192).

**Table 3** summarizes established biomarkers for predicting immunotherapy response in GC. PD-L1 expression (quantified by combined positive score (CPS)) is a validated predictor of response to PD-1/PD-L1 inhibitors, though its utility is limited by spatial heterogeneity and dynamic changes during therapy (193, 194). EBV positivity can reflect the expression of PD-L1 on GC cells, thereby predicting the sensitivity of patients to ICIs (194). MSI-High status identifies a subset with exceptional immunotherapy sensitivity due to high tumor mutational burden (195). MSI-H type GC has a high TMB, which can promote a strong immune cell response and is a useful biomarker for the efficacy of ICIs in treating GC (192). The circulating tumor DNA (ctDNA) dynamics enable real-time monitoring of treatment efficacy, where early clearance correlates with improved survival (196). While these biomarkers guide patient stratification, their predictive power is often suboptimal when used in isolation. Integration of PD-L1 CPS with TMB or MSI status may improve accuracy, yet prospective validation in multicenter cohorts is imperative. H. pylori infection can upregulate the expression of PD-1/PD-L1 in GC patients, which may indicate a poor prognosis (197). The gut microbiota reprograms the immune environment of TME by participating in innate and/or adaptive immunity, thereby regulating anti-tumor immunity and affecting the efficacy of ICIs (198).

**Table 3:** Classical biomarkers for predicting the response of GC to ICIs treatment.

Biomarker	Mechanism	Association with poor prognosis
PD-L1 (193, 194)	GC cells with high PD-L1 expression can form an immunosuppressive microenvironment by inducing T cell apoptosis or exhaustion, thereby promoting tumor growth and metastasis.	Positive correlation
EBV (194)	EBV-positive GC is associated with high CD8-positive T cell infiltration and PD-L1/L2 expression, suggesting its sensitivity to ICIs.	Positive correlation
TMB (192)	The higher the TMB, the better the clinical response to ICIs.	Negative correlation
ctDNA (196)	Dynamic ctDNA can serve as a potential biomarker for the immune therapeutic response in advanced GC, and the reduction of ctDNA is highly correlated with a better therapeutic response.	Positive correlation
MSI (195)	Patients with MSI-high are highly sensitive to PD-1/PD-L1 targeted immunotherapy.	Negative correlation
H. pylori (197)	H. pylori alters the immune microenvironment of GC, thereby influencing the efficacy of immunotherapy.	Negative correlation
gut microbiota (198)	The intestinal microbiome and its metabolites exert influence on the efficacy of immunotherapy by regulating innate immunity (including DC, macrophages and NK cells), adaptive immunity (involving CD8^+^ T cells and CD4^+^ T cells), and tumor cell immunogenicity.	Negative correlation

In recent years, significant progress has been made in biomarker research. Besides the well-known classical biomarkers, several molecular characteristics have been identified that are expected to be used for predicting the immune treatment response and prognosis of patients with GC. As summarized in **Table 4**, the emerging biomarkers - including METTL1, GNRI, PLR, NLR, AFF3, ALP, VSIG4, LSI, SERPINE1, SKAP1, CD103 and OX40+/LAG3+T cells - demonstrate significant potential in predicting the efficacy of ICIs in patients with GC. Additionally, another group of biomarkers (such as HIGD1B, CHAF1A, GGT5, AFP, NFS1, CLEC11A, ACVR1, TGFβ2, AKIRIN2, NPRL2, GPR176, CXCR4, COL5A2, GPX3, GBP2 and ITGB5) is associated with the prognosis of patients by regulating immune cell infiltration and remodeling the TME. KIF3C, and ITGB1 further delineate disease aggressiveness through their regulation of oncogenic signalling cascades. These discoveries underscore the potential of precision immunotherapy by leveraging biomarker-driven stratification in GC management. Beyond molecular biomarkers, gene-based prognostic models have been developed to enhance the prediction of treatment outcomes in GC.

**Table 4:** Novel biomarkers for predicting the response of GC to ICIs treatment.

Biomarker	Categories	Mechanism	Combined treatment	Drug resistance	Association with poor prognosis	Advantage	Limitation
METTL1 (210)	enzyme	METTL1 enhances the activity of GC cells by inhibiting T cell proliferation and upregulating the expression of CTLA4 and PDCD1.	Silent METTL1 can enhance the synergistic anti-tumor effect of anti-CTLA4 and anti-PD1 immunotherapies by down-regulating the expression of CTLA4 and PDCD1.	The tRNA m^7^G modification mediated by METTL1 affects drug resistance genes and can promote osimertinib resistance in non-small cell lung cancer cells (211). The role of METTL1 in GC resistance requires further investigation.	Negative correlation	1. Independent prognostic factor (Cox multivariate analysis). 2. Predict immune treatment sensitivity (model AUC > 0.85). 3. Guide combination therapy (CTLA4/PD1 inhibitors).	1. Tumor heterogeneity may affect the accuracy. 2. It requires large-scale clinical validation. 3. The mechanism has not been fully elucidated.
CHAF1A (212)	gene	The CHAF1A IHC score was positively correlated with the infiltration of NK cells and M1-type macrophages in EBV+GC.	By stratifying based on multiple indicators such as CHAF1A, EBV, MSI, TMB, and PD-L1, it is possible to precisely identify the population that benefits from immunotherapy and maximize the ORR of GC patients.	CHAF1A induces 5-FU resistance by upregulating the expression of thymidylate synthase (213).	Negative correlation	1. Independent prognostic factor (multi-cohort validation). 2. Enhance the predictive efficacy of existing markers (EBV/MSI/TMB/PD-L1). 3. Can be detected by IHC (clinical applicability).	1. The heterogeneity of EBV+GC has an impact. 2. There is a lack of mechanism research. 3. The sample size of the immunotherapy cohort is small.
GGT5 (214)	gene	GGT5 has a high positive correlation with immune-related genes; it promotes memory CD8^+^ T cells and the TME, and progresses.	Combining GGT5 inhibitors with ICIs can enhance the anti-tumor immune response by relieving the inhibitory effect of GGT5 on memory CD8^+^ T cells.	Downregulation of GGT5 expression can inhibit the chemotherapy resistance of lung adenocarcinoma cells (215). The role of GGT5 in drug resistance of GC needs further investigation.	Positive correlation	1. Independent prognostic factor (multi-cohort validation). 2. Predict ICI efficacy. 3. Drug development potential.	1. Requires large sample verification. 2. Mechanism is unclear.
AFP (216)	gene	AFP exerts its influence on immune function by inhibiting the functions of NK cells and DC.	Inhibiting AFP in combination with ICIs may enhance the therapeutic effect for AGC patients with elevated AFP levels.	AFP-producing gastric carcinoma, a highly malignant variant of GC, usually exhibits resistance to various chemotherapy drugs such as cisplatin (217).	Positive correlation	1. Serum detection is convenient (clinical popularization). 2. Independent predictor. 3. Subgroup of combined chemotherapy remains significant.	No verification of AFP expression in the tissue.
KIF3C (218)	gene	KIF3 regulates the PI3K-AKT signaling pathway to promote the progression of GC.	In the combined treatment with anti-PD-1 and anti-CTLA-4, patients with high expression of KIF3C showed higher immune treatment scores, suggesting that they are more likely to benefit from this type of combined immunotherapy.	KIF3C can induce chemotherapy resistance in multiple myeloma and lung squamous cell carcinoma (219, 220). The role of KIF3C in the drug resistance of GC needs further investigation.	Positive correlation	1. Independent prognostic factor. 2. Potential therapeutic target. 3. High diagnostic value.	Requires validation through animal models.
NFS1 (221)	enzyme	NFS1 affects the immune microenvironment and immunotherapy in GC patients.	Combining NFS1 inhibitors with anti-PD-1 or anti-CTLA-4 treatments can synergistically enhance the efficacy of GC immunotherapy by relieving the immune suppression mediated by the high expression of NFS1.	NFS1 is associated with platinum resistance. Inhibiting NFS1 can enhance the chemosensitivity of colorectal cancer to oxaliplatin (222). Whether NFS1 affects GC resistance requires further study.	Positive correlation	1. Independent prognostic factor. 2. Predict immune treatment efficacy (the low expression group of PD-1/CTLA4 is more sensitive). 3. High diagnostic value.	1. Lack of *in vivo* and *in vitro* validation. 2. Variability in immune therapy response.
CLEC11A (223)	gene	Down-regulation of CLEC11A enhances the infiltration of cytotoxic CD8 T cells and helper CD4 T cells in tumors, while reducing the proportions of M2 macrophages, MDSCs and Tregs.	Combining CLEC11A inhibitors with anti-PD-1 or anti-CTLA-4 therapy can significantly enhance the therapeutic effect.	CLEC11A is involved in genomic instability and epigenetic modifications, and may be related to drug resistance. Further studies are needed to confirm this.	Positive correlation	1. Independent prognostic factor. 2. Predict immune treatment efficacy. 3. Build a 6-gene immune risk model.	1. Clinical translation requires prospective trials. 2. The specific related mechanisms have not been clarified.
ACVR1 (224)	receptor	ACVR1 regulates the TIME by increasing the content and composition of the TME matrix and enriching the related pathways, promoting cancer progression, thereby facilitating the progression of GC.	Combining ACVR1-targeted anti-treatment with drugs such as paclitaxel and erlotinib, or exploring ICIs other than PD-1/CTLA-4, is expected to overcome the limitations of conventional immunotherapy responses in patients with high ACVR1 expression in GC.	ACVR1 can induce the resistance of acute myeloid leukemia cells to FLT3 inhibitors (225). However, whether ACVR1 leads to resistance in GC cells remains to be investigated.	Positive correlation	1. Independent prognostic factor. 2. Guide combined treatment (chemotherapy sensitive). 3. Predict immune treatment resistance.	1. Lack of *in vivo* and *in vitro* validation. 2. The downstream signaling targets have not been clearly identified.
GNRI (226)	index	1. When there is hypoalbuminemia, the entire body is in an inflammatory state, which inhibits the immune function. 2. Weight loss and insufficient energy reserves weaken the immune system function.	The combined assessment with GNRI and ICIs treatment can precisely identify patients with good nutritional status, thereby accurately screening out the population that can benefit from GC immunotherapy.	Further study is needed.	Negative correlation	1. Calculation is simple (only requiring albumin + body weight). 2. Cost is low.	1. The heterogeneity of ICI drugs was not taken into account. 2. It is necessary to combine inflammatory indicators such as C-reactive protein.
PLR (227)	index	The elevated PLR in GC patients treated with ICI is significantly associated with the deterioration of OS and PFS.	The combined PLR assessment in combination with ICIs treatment can precisely identify patients with lower PLR, thereby accurately screening out those who are more likely to have a better prognosis for GC immunotherapy.	Further study is needed.	Positive correlation	1. Platelet aggregates enhance tumor immune escape. 2. Decreased lymphocytes lead to an insufficient immune response.	Requires verification through forward-looking research.
NLR (228)	index	The higher NLR of GC patients treated with ICIs is associated with poorer OS and worse ORR	The combined NLR assessment with ICIs treatment can precisely identify patients with lower NLR, thereby accurately screening out the population with better prognosis and higher objective response rate for GC immunotherapy.	Further study is needed.	Positive correlation	Easy testing.	Not included in the irAE data.
AFF3 (229)	gene	AFF3 affects the efficacy of ICIs by promoting the expression of TIICs and ICs in the TME.	Targeted inhibition of AFF3 combined with anti-PD-1/CTLA-4 therapy is expected to improve the immune treatment response of GC patients with low TMB/MSI by reversing the high expression state of immune checkpoint molecules mediated by AFF3.	The overexpression of AFF3 leads to the resistance of breast cancer cells to tamoxifen (230). However, the role of AFF3 in the drug resistance of GC cells is not yet very clear.	Positive correlation	1. Independent prognostic factor. 2. Predicting response to immunotherapy.	Experimental verification is required for the molecular mechanism.
TGFβ2 (231)	antibody	TGFβ2 is significantly positively correlated with infiltration of various immune cells, cytokines, stemness markers, interstitial scores and EMT.	Combining the TGFβ2 inhibitor with anti-PD-1/PD-L1 or anti-CTLA-4 immunotherapy can enhance the efficacy of GC immunotherapy by simultaneously blocking parallel immune suppression pathways, and is particularly suitable for patients with high TGFβ2 expression.	TGFβ2 can activate the PI3K-AKT pathway through mesenchymal stromal cells, thereby promoting drug resistance in gastrointestinal stromal tumors (232).	Positive correlation	1. Independent prognostic factor. 2. Predicting high response to ICB.	Biological experiments are needed for verification.
AKIRIN2 (233)	gene	The increase in AKIRIN2 expression is associated with poor prognosis of CD8 T cells and CD4 memory T cells, as well as elevated levels of immune infiltration.	Combined chemotherapy (cisplatin/paclitaxel/5-FU) and anti-PD-1 immunotherapy can synergistically enhance the therapeutic response in patients with high AKIRIN2 expression in GC.	In chronic myeloid leukemia, the imatinib-resistant K562 cells exhibit an increase in AKIRIN2 protein levels in the nucleus (234). The role of AKIRIN2 in GC resistance requires further investigation.	Negative correlation	1. Independent prognostic factor. 2. Simultaneously predicting response to chemotherapy and immunotherapy.	The functional state of CD8^+^ T cells has not been verified.
NPRL2 (235)	gene	NPRL2 affects the infiltration level of immune cells.	Targeting the high expression state of NPRL2 in combination with ICIs therapy is expected to enhance the immune response rate of STAD patients by optimizing the tumor immune microenvironment.	In STAD cells, the expression of NPRL2 is positively correlated with the drug sensitivity to 5-FU and paclitaxel.	Negative correlation	Independent prognostic factor.	High methylation of the promoter leads to expression deficiency.
ALP (236)	enzyme	The ALP level > 225 U/L is associated with poor response to ICIs and shorter PFS in HER2-negative mGC patients.	Incorporating ALP testing into the pre-treatment evaluation system for HER2-negative metastatic GC patients, with a screening threshold of ≤ 225 U/L, helps to accurately identify the potential beneficiaries of ICIs treatment.	ALP is involved in the processes of phosphorylation/dephosphorylation, altering the pH value of the tumor microenvironment and the recruitment/polarization of immune cells, thereby promoting immune evasion (237). However, the role of ALP in GC resistance still requires further study.	Positive correlation	1. Convenient detection. 2. Independent prognostic factor.	1. Low specificity. 2. Requires prospective research for validation.
VSIG4 (238)	protein	VSIG4 might be a “secondary” immune checkpoint molecule in the gastric immune microenvironment.	Combined anti-VSIG4 targeted therapy, when combined with existing ICIs, is expected to enhance the efficacy of GC-specific immunotherapy by simultaneously blocking two immune-suppressive pathways.	The resistance to immunotherapy with Ipilimumab/Nivolumab in VSIG4 and clear cell renal cell carcinoma is closely related (239). This provides a reference for studying the role of VSIG4 in drug resistance of GC.	Positive correlation	1. Independent prognostic factor. 2. GC specificity.	1. Requires tissue biopsy for detection. 2. Has not yet been applied in clinical practice.
LSI (240)	index	The LSI is negatively correlated with OS, PFS, and DCR of GC patients treated with PD-1 inhibitors. The tumors with higher baseline LSI are more invasive. The number and percentage of circulating lymphocytes in the LSI group are significantly decreased, and cellular immunity is impaired.	The combined serum iron level test and anti-PD-1 immunotherapy can be used to identify patients with LSI and conduct risk stratification, guiding them to receive combined iron supplementation or other auxiliary treatments to improve the immune therapy response.	Further study is needed.	Positive correlation	1. Convenient detection. 2. Dynamic monitoring.	Non-specific, susceptible to chemotherapy.
SERPINE1 (241)	gene	The SERPINE1 high-expression group includes immune cells such as CD4^+^ T cells, B cells, CD8^+^ T cells, macrophages, and neutrophils, which express at a higher level. It also has a synergistic effect with immune checkpoints PD1 and PD-L1.	Combining SERPINE1 inhibitors with anti-PD-1/PD-L1 immunotherapy can achieve this by simultaneously blocking the positive regulatory association between SERPINE1 and immune checkpoints, reversing the immunosuppressive microenvironment, and enhancing the efficacy of GC immunotherapy.	Silencing SERPINE1 enhances the sensitivity of GC xenografts to anti-PD-1 therapy (242).	Positive correlation	The specificity of GC is high.	It requires multi-center validation.
GPR176 (243)	receptor	GPR176 is significantly correlated with various immune checkpoint genes in GC, and it influences the GC process by regulating immune cells.	Combining GPR176-targeted inhibition with anti-CTLA4/PD1 immunotherapy can reduce the risk of tumor immune escape in patients with high GPR176 expression in GC, and synergistically enhance the efficacy of immunotherapy.	GPR176 can induce chemotherapy resistance in esophageal cancer cells (244). However, the role of GPR176 in gastric cancer resistance remains unclear.	Positive correlation	1. High diagnostic efficacy. 2. Independent prognostic value.	The protein level needs to be verified for clinical applicability.
CXCR4 (245)	receptor	The CXCR4 overexpressed group was significantly enriched in helper T cells, cytotoxic T cells, B lymphocytes, monocytes and CTLA4 pathways and participated in the remodeling of the immune microenvironment of GC.	Combining CXCR4 inhibitors with ICIs therapy can reverse the immunosuppression mediated by high CXCR4 expression and synergistically enhance the efficacy of immunotherapy in GC patients.	CXCR4 inhibits autophagy in cells, thereby promoting the chemotherapy resistance of GC cells to 5-FU (246).	Positive correlation	Pan-cancer validation.	Multi-center validation is needed.
SKAP1 (247)	protein	SKAP1 restores the anti-tumor immune response and promotes immune-mediated elimination of malignant cells by inhibiting multiple immune checkpoints.	Combining SKAP1 inhibitors with multiple immune checkpoint inhibitors such as anti-PD-1, anti-TIM-3, and anti-LAG3, through synergistic inhibition of the upregulation of immune checkpoints mediated by SKAP1, can reverse immune therapy resistance.	SKAP1 plays a significant role in regulating the sensitivity of pancreatic adenocarcinoma cells to chemotherapeutic drugs (248). The role of SKAP1 in drug resistance to GC needs further investigation.	Positive correlation	1. High diagnostic efficacy. 2. Targeted therapy (JAK/PI3K inhibitors).	Blood testing technology needs to be developed.
COL5A2 (249)	gene	The expression of COL5A2 is significantly correlated with the macrophage M2 gene markers as well as the cell markers of helper T cells (Th1, Th2). It may promote immunosuppression by enhancing Tregs differentiation and T cell exhaustion.	High expression of COL5A2 is positively correlated with immune checkpoint molecules and predicts immune therapy resistance. Combining the use of COL5A2 inhibitors with anti-CTLA-4/PD-1 therapy is expected to synergistically enhance the GC immune response.	COL5A2 enhances the resistance of head and neck squamous cell carcinoma cells to erlotinib. The role of COL5A2 in chemotherapy resistance of GC requires further investigation.	Positive correlation	Predicting immune therapy response.	Requires verification through forward-looking research.
GPX3 (250)	enzyme	GPX3 contributes to GC by increasing the infiltration of tumor immune cells and enhancing the expression of immune checkpoints.	Based on the expression level of GPX3, patients with GC are stratified. Those with low expression are more suitable for ICIs combined with chemotherapy, while those with high expression need to be treated with GPX3-targeted therapy to enhance the synergistic efficacy of immunotherapy and chemotherapy.	GXP3 can induce the drug resistance of GC cells to chemotherapy drugs such as 5-FU and doxorubicin.	Positive correlation	Predicting chemotherapy sensitivity.	More basic research and clinical trials are needed.
GBP2 (251)	enzyme	GBP2 is associated with an immune hot TME in GC.	Combining GBP2 expression assessment with ICIs treatment can precisely identify patients with high GBP2 expression in GC, thereby accurately screening out the immunotherapy-advantaged population characterized by PD-L1 positivity, abundant CD8^+^ T cell infiltration, and dMMR enrichment.	GBP2 inhibits the growth of colorectal cancer cells by interfering with the Wnt signaling pathway and enhances the sensitivity to paclitaxel (252). However, the role of GBP2 in the resistance of GC cells still needs to be studied.	Positive correlation	Pan-cancer application.	The GBP2-STAT1 axis needs further validation.
ITGB1 (253)	receptor	The expression of ITGB1 is associated with a poor response to immunotherapy and is related to some immunosuppressive factors in the GC environment. It activates the Wnt/β-catenin signaling pathway.	Patients with GC who have high expression of ITGB1 have poor responses to ICIs due to the decreased TMB and the upregulation of PD-L1/TIM3. Combining ITGB1-targeted inhibition with anti-PD-1/CTLA-4 therapy is expected to have a synergistic effect.	The activation of the ITGB1/BCL9L/β-catenin signaling pathway can promote the progression of GC and drug resistance (254).	Positive correlation	Predicting immune therapy response.	Real-world data are required to verify the predictive value of immunotherapy.
CD103 (255)	antibody	High expression of CD103 in PD-1 ^+^ CD8 ^+^ T cells of patients with advanced GC after nivolumab treatment for 2 weeks	Joint monitoring of CD103^+^PD-1^+^CD8^+^ T cells can predict the efficacy of anti-PD-1 therapy, and combined expansion of this cell population is expected to overcome immune therapy resistance.	Restoring the quantity and function of CD103 DC can reactivate cross-resistant tumors to become sensitive to immunotherapy (256). The role of CD103 in the drug resistance of GC cells requires further investigation.	Positive correlation	Non-invasive peripheral blood test.	A large sample size is needed to validate the critical value.
OX40/LAG3 (257)	protein	Nivolumab treatment enhances central/effector memory and activation of CD4/CD8 T cell effector subsets. The expression levels of LAG-3 and OX40 on T cells are related to the efficacy of nivolumab. The proportions of OX40 and LAG3-positive T cells in peripheral blood before and after anti-PD-1 treatment are different.	The combined monitoring of LAG3^+^ and OX40^+^ T cells in conjunction with anti-PD-1 treatment can precisely predict the efficacy of immunotherapy in GC patients, providing a screening basis for the combined targeting of LAG3/OX40 pathways.	The anti-LAG3 antibody relatlimab, combined with PD-1 and chemotherapy, is currently being studied in AGC (NCT03044613, NCT04062656 and NCT02935634) (258). In colorectal cancer, OX40 can reverse immune escape and reverse drug resistance (259). The role of OX40 in GC resistance requires further investigation.	Positive correlation	High combined predictive efficacy.	Detection costs are high. The mechanism of synergistic effects with PD-1 needs to be explored.
ITGB5 (260)	receptor	The overexpression of ITGB5 is strongly positively correlated with the infiltration levels of macrophages and monocytes, significantly influencing the immune response.	Combined detection of ITGB5 expression and ICIs treatment: Individuals with low expression can directly benefit, while those with high expression need to undergo targeted intervention with ITGB5 to enhance the immune response.	ITGB5 upregulates CSNK1A1, thereby disrupting the EPS15/EGFR complex, leading to the development of resistance to sorafenib in hepatocellular carcinoma *in vivo* (261). The role of ITGB5 in GC resistance requires further investigation.	Positive correlation	Serum testing is non-invasive. Combined diagnosis with CA19–9 enhances diagnostic value.	The efficacy of a single diagnosis is limited.

### 4.1 Gene model based on cellular functional characteristics/cell death

Cell-function-centric gene signatures are now being deployed to refine immunotherapy prediction in GC. Sun et al. established a 12-gene NK cell-associated signature (NKCAS), stratifying GC patients into low-risk groups (LRG) and high-risk groups (HRG). Correlation analyses revealed that LRG patients exhibited higher PD-L1 mRNA expression and TMB, along with a lower TIDE score, suggesting enhanced responsiveness to ICIs therapy (199). TAMs, the most prevalent immune cells in the TME, have also been leveraged for risk stratification. Xin et al. developed a TAM-related prognostic signature based on nine genes, demonstrating that lower TAMs scores correlate with improved prognosis and superior immunotherapy outcomes (200). Additionally, CD8^+^T cells play a critical role in antitumor immunity by secreting effector cytokines and cytotoxic granules to target cancer cells. Using single-cell RNA sequencing (scRNA-seq), Li et al. identified eight CD8^+^ T cell-associated genes and constructed a prognostic model, revealing that HRG patients exhibited reduced sensitivity to immunotherapy (201). These immune-cell-centric models underscore the need for prospective optimisation and clinical validation.Beyond adaptive immunity, programmed cell death modalities shape immunogenicity. Liu et al. proposed a PANoptosis-related risk score (PANS) based on genes regulating PANoptosis, a form of inflammatory programmed cell death. Low-PANS patients displayed higher TMB and MSI, reduced tumor purity, and increased sensitivity to immunotherapy, correlating with improved prognosis (193).

### 4.2 Gene model based on metabolic/apparent genetic processes

Recent studies have expanded the scope of biomarker discovery by screening key genes involved in diverse biological processes, including cellular structure, metabolic pathways, epigenetic regulation, and programmed cell death, to construct predictive models for immunotherapy response in GC.

Liu et al. identified Golgi apparatus-related genes (GARGs) and developed a risk stratification model based on five GARGs. Using the median risk score as a cutoff, GC patients were classified into HRG and LRG, with the latter exhibiting enhanced immunoreactivity and higher TMB (202). Similarly, Ma et al. established a risk-scoring model using 10 differentially expressed immune-related genes (DEIRGs) and demonstrated that LRG patients had elevated TMB and immunophenotype score (IPS), suggesting greater immunotherapy sensitivity (203). Metabolic reprogramming also plays a critical role in shaping immunotherapy outcomes. Glycolytic reprogramming was interrogated by Meng et al., whose five-gene glycolysis index revealed that high glycolysis associates with reduced neoantigen load and TMB, poorer OS and paradoxically higher MSI incidence (204).In the context of epigenetic regulation, Yin et al. derived a prognostic m6Ascore based on five m6A-related genes. IPS analysis indicated that patients with higher m6Ascores showed superior therapeutic responses to CTLA-4 and/or PD-1 blockade (205). Collectively, these multi-omics models underscore that GC immunogenicity is orchestrated by convergent metabolic and epigenetic pathways, necessitating integrative scoring systems and combination regimens designed from a systems-biology perspective.

### 4.3 Clinical indicators and imaging integration model

Building upon the genetic and multi-level biological understanding of immune responses in GC, researchers have extended their investigations to readily available clinical indicators and imaging features, developing more clinically applicable predictive models. Zuo et al. constructed a GC immune prognosis score incorporating white blood cell count, lymphocyte count, and the international normalized ratio (INR). Their findings demonstrated that GCIPS effectively predicts outcomes in GC patients undergoing ICIs therapy, with higher scores correlating with shorter PFS and OS, indicative of poorer prognosis (206). Similarly, Zhan et al. developed and validated a CT-based radiomic biomarker to assess MSI status and immunotherapy response. Patients classified into the low rad-score group exhibited significantly prolonged median PFS and OS compared to those in the high rad-score group, suggesting superior therapeutic efficacy in the former cohort (207). Further advancing this field, Sun et al. established two CT-derived imaging biomarkers—lymph imaging biomarker score (LRS) and bone marrow imaging biomarker score (MRS)—and demonstrated that patients with high LRS or low MRS derived greater benefit from immunotherapy (208).

These studies underscore the clinical utility of integrated clinical-imaging models in predicting immunotherapy outcomes for GC, offering quantifiable, non-invasive tools for risk stratification. Such models hold promise for identifying potential responders, guiding personalized treatment decisions, and mitigating resource waste and patient burden associated with ineffective therapies. Future efforts should focus on standardizing these models, validating them through multicenter collaborations, and elucidating their underlying mechanisms to facilitate their translation from research tools into clinical practice, ultimately enabling precision immunotherapy stratification.

### 4.4 Matrix microenvironment-driven model

Cancer-associated fibroblasts (CAFs) represent a predominant stromal component within the TME and play a pivotal role in modulating tumor progression and therapy resistance. Xia et al. developed a CAF-derived angiogenesis prognostic score (CAPS) system to stratify the clinical outcomes of GC patients undergoing immunotherapy. Their findings demonstrated that patients with high CAPS exhibited a heightened propensity for immune evasion and derived diminished therapeutic benefit from immunotherapy compared to the low CAPS cohort (209). By quantifying the pro-tumorigenic mechanisms of CAFs—including angiogenesis and immunosuppression—the CAPS system provides a clinically actionable, TME-based prognostic tool. Importantly, its predictive capacity may facilitate the identification of immunotherapy-responsive patients, thereby optimizing treatment selection and mitigating unnecessary adverse effects and economic burdens associated with ineffective therapies.

## 5 Conclusion and perspectives

Immunotherapy has rapidly evolved into a cornerstone of advanced GC management, complementing surgery, chemotherapy, radiotherapy, and targeted agents. Despite the demonstrable activity of ICIs, adoptive cell therapy, cancer vaccines, and CAR-T cells, modest response rates and immune-evasion pathways continue to limit broad clinical impact. Future strategies, therefore, converge on two axes: (1) rational combination regimens that integrate immunotherapy with surgery, radiotherapy, chemotherapy or novel ICIs, and (2) precision immuno-oncology platforms that exploit nano-delivery systems, microenvironment-targeted adjuvants and TCM derivatives. Central to these efforts is biomarker-guided patient selection. PD-L1 CPS remains the most widely adopted clinical metric, yet spatial heterogeneity and temporal dynamics demand complementary tools. Liquid-biopsy-based ctDNA kinetics, multi-omic signatures (e.g., METTL1, HIGD1B), and imaging-derived radiomic scores are now being prospectively validated in multicenter trials to refine responder identification and monitor on-treatment adaptation. Ultimately, iterative biomarker discovery and large-scale clinical verification will be required to translate these advances into individualized, safer and more effective GC immunotherapy algorithms.
